# Hemoglobin Titusville: a rare low oxygen affinity hemoglobinopathy

**DOI:** 10.1002/ccr3.941

**Published:** 2017-05-11

**Authors:** Senan John Yasar, Vivian Irene Ravn Berg, Asim Ahmad, Donald Doll

**Affiliations:** ^1^ University of MissouriOne Hospital Drive 29Columbia65212MissouriUSA; ^2^ Poznan University of Medical Sciences41 Jackowskiego StPoznan60‐512Poland

**Keywords:** dyspnea, hemoglobin Titusville, hemoglobinopathy, hypoxia, oximetry

## Abstract

The relevance of this case lies in the extensive diagnostic workup that can be avoided with proper laboratory evaluation of relatively unsophisticated tests.

There are hundreds of hemoglobin variants involving genes from both the alpha and beta gene clusters. When mutations alter the normal hemoglobin tetramer of two alpha and two beta chains, hemoglobin's function to bind oxygen and carbon monoxide may be compromised or enhanced. Although not all variants of hemoglobin may cause clinical pathology, some may be associated with distinct symptoms of disease. We describe a patient with exercise intolerance and syncope with hemoglobin Titusville, a low oxygen affinity variant hemoglobin.

A 24‐year‐old white Russian female nonsmoker was initially evaluated by her primary care physician for a history of syncope and exercise intolerance and was eventually referred to cardiology for low pulse oximetry readings on multiple visits. SpO2 was found to be in the range of 89–90%. Echocardiogram was performed which showed a small patent foramen ovale which was considered hemodynamically insignificant. In addition, patient's ABG revealed pH 7.41, PaO2 104 mmHg, pCO2 40 mmHg, and base excess of 0.7 mmol/L. These results excluded arterial hypoxemia. Due to these abnormal results, the patient was referred to hematology for concern of a possible hemoglobinopathy. She had no prior family history of hemoglobinopathy. Examination was unremarkable, of note there was no cyanosis. Initial laboratories revealed a hemoglobin of 11.5 g/dL, a WBC count of 3.3 × 109/L, and a methemoglobin level of 1.8%. Hemoglobin electrophoresis demonstrated a hemoglobin A component comprising 81.1%, hemoglobin A2 component comprising 2.3%, hemoglobin F component comprising 0.6%, as well as a variant hemoglobin comprising 16.0%. Molecular studies revealed a DNA change on codon 94 with GAC to AAC, resulting in an amino acid substitution of aspartic acid to asparagine, which is diagnostic of hemoglobin Titusville.

Hemoglobin Titusville was initially described in 1975 in a healthy 3‐year‐old African American female during a screening program for hemoglobinopathies 1. According to Luo et al., this hemoglobin variant occurs due to a mutation of the a2‐globin gene codon 94 of GAC to AAC, or aspartic acid to asparagine. This amino acid substitution is clinically relevant due to the fact that the wild‐type aspartic acid at codon 94 normally interacts with asparagine on codon 102 which occurs at the *α*1*β*2 interface 2. In 2014, a 19‐year‐old Israeli soldier with significant smoking history and obstructive lung disease was described to have presented with cyanosis as well as hypoxemia 3. In addition, a report was published of three family members of Swedish and Finish descent whom presented with low oxygen saturation as well as cyanosis, confirmed to be hemoglobin Titusville with *α*‐globin gene amplification via PCR. Lastly, a 14‐year‐old Caucasian male with 17% hemoglobin Titusville documented by DNA sequencing was noted 2. His Hemoglobin was 14.6 g/dL with a blood oxygen saturation of 83%.

Our patient presented with syncope and exercise intolerance, and evaluation showed low oxygen saturation with normal/slightly elevated oxygen level on ABG. It is unclear as to the exact etiology of the patient's slightly elevated oxygen level as her ABG was taken on room air and did not support a respiratory alkalemia type picture as would be expected had the patient been hyperventilating. Such discordance between SpO2 and PaO2 should alert clinicians to consider the diagnosis and forgo an extensive evaluation for cardiopulmonary conditions. In the case of the young Israeli soldier reported by Marcus et al., further diagnostic workup including CT, TTE, TEE, CT‐angiography, as well as stress testing before a hemoglobinopathy, was considered.

When presented with a case of hemoglobinopathy, it is also important to note the degree of anemia which may not always correlate with the severity of symptoms. For example, in cases of high oxygen affinity hemoglobinopathies, such as Hb Chesapeake, one of the prominent features of the disease includes erythrocytosis. Despite this, patients are still starved of oxygen at the cellular level, due to an inability for hemoglobin molecules to dissociate from oxygen. In such cases, the P50 is low. On the other hand, low oxygen affinity hemoglobinopathies are often associated with an increase in P50. Patients with a moderately right‐shifted oxygen equilibrium curve may be mildly anemic, but some patients with very right shift curves are not anemic. Nonetheless, hemoglobin will have the tendency to dissociate from oxygen early, thus causing tissue hypoxia and cyanosis. With this considered, it may be theorized that this tissue hypoxia can serve as a source of increased drive for erythropoietin production. This would in turn explain why our patient only suffered from a mild degree of anemia (it should be noted that erythropoietin levels were never measured in our subject). It has also been speculated that this decreased affinity for oxygen may in fact serve as a beneficial feature that makes oxygen unloading and delivery to tissue more efficient. This theory has support in animal models; indeed, mice, whom were engineered with the mutation Asp94Asn, were found to have double the exercise tolerance of those without the mutation 4. Of note our patient presented with exercise intolerance. Although HPLC and measuring P50 may be helpful in the differential diagnosis of patients with unexplained cyanosis and low oxygen saturation, globin gene sequencing is the definitive diagnostic test for low oxygen affinity hemoglobins 5.

In conclusion, hemoglobin Titusville is a rare, inherited defect of hemoglobin structure that is most commonly found in individuals of northern European background 3. The molecular basis of this disorder is an amino acid change on codon 94 with GAC to AAC. Although this disorder is rare, and no treatment is indicated, it is important to make an early diagnosis to avoid unnecessary and expensive evaluation and to alleviate anxiety for the patient.

## Authorship

SJY: provided the case from faculty member Dr Doll and created the majority of the manuscript content. VIRB: under the instruction of Dr Yasar helped to construct the content for discussion as well as significant contribution of writing. AA: guided Dr Yasar and provided edits to ensure manuscript was novel and a clinical take‐home point was addressed. DD: the first member of team to come across case and presented it as a topic for a case report. Oversaw and edited the manuscript.

## Conflict of Interest

None declared.
